# Evaluating the Impact of Goal Setting on Improving Diet Quality in Chronic Kidney Disease

**DOI:** 10.3389/fnut.2021.627753

**Published:** 2021-03-12

**Authors:** Chi H. Chan, Marguerite Conley, Marina M. Reeves, Katrina L. Campbell, Jaimon T. Kelly

**Affiliations:** ^1^Faculty of Health Science and Medicine, Bond University, Gold Coast, QLD, Australia; ^2^Department of Nutrition and Dietetics, Princess Alexandra Hospital, Brisbane, QLD, Australia; ^3^Faculty of Medicine, School of Public Health, The University of Queensland, Brisbane, QLD, Australia; ^4^Allied Health Services, Metro North Hospital and Health Service, Brisbane, QLD, Australia; ^5^Centre of Applied Health Economics, Griffith University, Brisbane, QLD, Australia; ^6^Menzies Health Institute Queensland, Griffith University, Gold Coast, QLD, Australia

**Keywords:** chronic kidney disease, diet quality, goal setting, self-managament, telehealth

## Abstract

**Background:** Improving diet quality in chronic kidney disease (CKD) is challenging due to a myriad of competing recommendations. Patient-centered goal setting can facilitate dietary behavior change; however, its role in improving diet quality in CKD has not been investigated.

**Aim:** The aim of the study is to evaluate the effects of goal setting on improving diet quality in stages 3–4 CKD.

**Methods:** Forty-one participants completed a 6-month dietitian-led telehealth (combined coaching calls and text messages) intervention as part of a larger RCT. Participants set one to two diet-related SMART goals and received weekly goal tracking text messages. Dietary intake was assessed using the Australian Eating Survey at baseline, 3, and 6 months, with diet quality determined using the Alternate Healthy Eating Index (AHEI).

**Results:** Significant improvements in AHEI (+6.9 points; 95% CI 1.2–12.7), vegetable (+1.1 serves; 95% CI 0.0–2.3) and fiber intake (+4.2 g; 95% CI 0.2–8.2) were observed at 3 months in participants setting a fruit and/or vegetable goal, compared with those who did not. However, no significant or meaningful changes were observed at 6 months. No other goal setting strategy appeared in effect on diet intake behavior or clinical outcomes in this group of CKD participants.

**Conclusions:** Patient-centered goal setting, particularly in relation to fruit and vegetable intake, as part of a telehealth coaching program, significantly improved diet quality (AHEI), vegetable and fiber intake over 3 months. More support may be required to achieve longer-term behavior change in stages 3–4 CKD patients.

## Introduction

Chronic kidney disease (CKD) is a public health issue affecting over 10% of the global population (1). Dietary intake and lifestyle behaviors are important elements in the self-management of CKD and its associated comorbidities (2, 3). Diet recommendations have historically focused on restricting nutrients such as protein, sodium, potassium, and phosphate to slow the progression of CKD and manage its associated conditions such as hyperkalemia and hyperphosphatemia. However, the evidence base for these nutrient restrictions is conflicting, and people with CKD struggle to adhere to these recommendations for a long term (4). Recent evidence has hypothesized that food-based dietary pattern approaches may improve CKD outcomes (4). Observational data have shown that a healthy dietary pattern is associated with decreased risks of all-cause mortality in people with CKD (5) and reduces risks of CKD comorbidities such as cardiovascular disease and diabetes mellitus (6, 7). A small number of randomized controlled trials (RCTs) have also shown that people with CKD can improve their diet quality in the short term (8). However, the long-term adherence to diet pattern interventions is unknown and remains a critical factor to address in helping patients succeed in changing their dietary behavior in practice for a long term (9–11).

Goal setting as a strategy for behavior change has been shown to be effective in CKD comorbidities management and may facilitate long-term behavior change through improved self-efficacy (12, 13). For example, in diabetes and heart failure patients, goal setting has been found to be beneficial for behavioral changes in dietary intake and physical activity (14), and improves self-efficacy and self-management skills (15, 16). People who set a specific, measurable, achievable, realistic, and timed (SMART) goal are likely to attain a successful outcome (17), and it is hypothesized that this might help people with CKD to achieve better dietary intake. However, the effect of SMART goal setting on improving diet quality in CKD-specific populations has not been evaluated to date.

A recent pilot RCT of a telehealth coaching program in people with stages 3–4 CKD showed significant and meaningful improvements in diet quality following 3 months of intensive coaching (8, 18). However, these effects were attenuated after 3 months of less-intensive coaching. As coaching was tailored to participant goals in this study, this has prompted the need to investigate whether specific goal setting influenced the change in diet quality in this sample of individuals with CKD. Therefore, this current study aimed to investigate whether specific dietary goal setting was associated with improvements in diet quality and other dietary and health indicators in people with stages 3–4 CKD.

## Methods

This study is a single-arm secondary analysis of intervention participants recruited from the previous ENTICE-CKD trial. The previous ENTICE-CKD study was a pilot RCT originally conducted in 80 participants (*n* = 41 intervention; *n* = 39 control) across three sites with stages 3–4 CKD (eGFR <60 ml/min/1.73 m^2^), who owned a mobile phone (18). All participants were metabolically stable and were cleared to participate by their treating nephrologists. Each participants' nephrologist maintained responsibility for the patients' medical management throughout the study period. A single-arm analysis within the intervention group was the most appropriate to answer our specific research aim as the control group participants in the previous ENTICE-CKD study did not receive any intervention to set SMART goals nor had data collected on goal achievement throughout the study. This study conformed to the Declaration of Helsinki; all participants provided written informed consent, and the research protocol was approved by the Metro South Hospital and Health Service and Bond University Human Research Ethics Committees.

**Figure 1:**
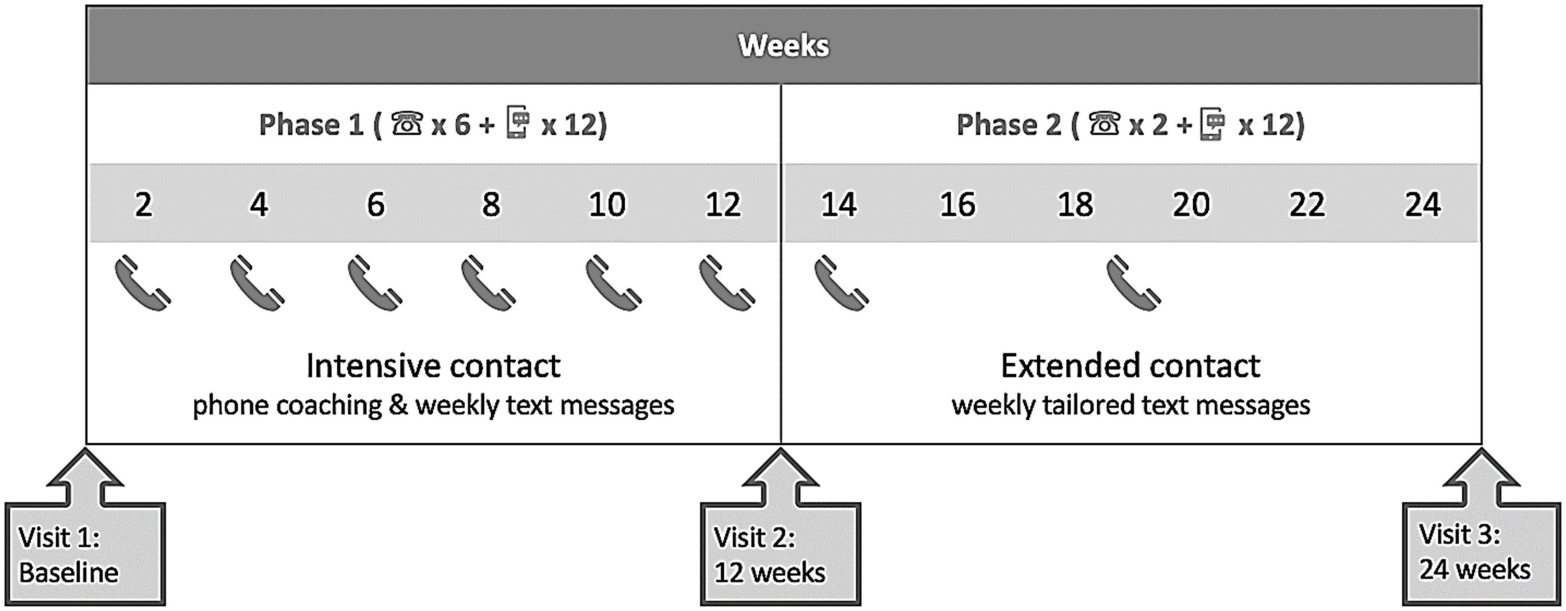
Study scheme showing the intervention approach used in the ENTICE-CKD study.

The previous ENTICE-CKD intervention is described in detail elsewhere (16, 18). Briefly, the previous ENTICE-CKD study was a telehealth intervention completed in two phases, phase 1 (baseline to 3 months) and phase 2 (3–6 months). Figure 1 shows the study schema; the intervention participants received intensive telehealth coaching (telephone and text messages) fortnightly from the same Accredited Practicing Dietitian (APD), with weekly tailored text messages to support the coaching content in phase 1. Each coaching call addressed improving diet quality in line with the Australian Dietary Guidelines (19), which was further tailored to suit individual participant-associated comorbidities and their set goals. A program workbook was provided to all participants, which included sections relevant to SMART goal setting strategies and self-monitoring practice. In the initial coaching call, the dietitian coach guided participants through the workbook and worked with participants to set one to two diet-related SMART goals. The setting of each SMART goal was done collaboratively between the dietitian and the participant, based on a comprehensive medical and nutrition assessment and the participant stage of change. The purpose of the intervention was to improve diet quality, and therefore, each SMART goal set by participants was designed to address an area of dietary intake in order to achieve this, rather than the improvement in a clinical outcome. The dietitian provided evidence-based nutrition counseling using motivational interviewing techniques (18), addressing barriers to goal achievement, and action planning for the following 2 weeks. Participants also determined their individual preference for the timing and frequency of receiving text messages, which supported the coaching content and reminded participants of their set goals (detailed in Table 1). In the five subsequent coaching calls, the dietitian checked participants' progress in regard to their set goals and provided counseling support as per the study protocol (18). In phase 2, the intervention participants received no further coaching calls, however, and were continued to be sent the same tailored text messages at a frequency determined by the participants (protocol detailed in Table 1).

**Table 1:** Text message type and examples.

**Message type**	**Example text**	**Frequency**
Educational	Dietary fiber intake reduces your cholesterol levels and controls your blood sugar. Include whole grain bread and cereals, fruits and vegetables regularly	1–4 every 2 weeks
Self-monitoring	Hi, (name). Are you keeping track of your fruit/vegetable intake every day? Remember your goal to meet at least 5 serves this week	1–4 every 2 weeks
Goal check	Hi, (name). Did you reach your goal to eat 5 fruits/vegetables 4 times this week? Text me back yes or no to let me know	2–4 every 2 weeks
Educational permutations	Choose high-fiber, low-potassium breakfast cereals. Good choices are Multigrain Weetbix, Rolled Oats, Guardian, Oatbritz, and Special K	0–2 every 2 weeks

The tailored text messages in both phases were sent to individual participants using a web-based, semi-automated text message management platform (Propelo, www.propelo.com.au), developed and administered by The University of Queensland's School of Public Health (20). Tailored text messages were used to remind and track participants' goals each week (see Table 1). Participants were asked to text back either “yes” or “no” regarding their weekly goal achievement.

Data were collected at three time-points throughout the study including baseline, end-of phase 1 (3 months), and end-of phase 2 (6 months). The primary outcome was the change in the Alternate Healthy Eating Index (AHEI)-2010. The AHEI-10 score is derived from the consumption of 11 food groups or nutrients: (1) vegetables, (2) fruits, (3) whole grains, (4) sugar-sweetened beverages and fruit juice, (5) nuts and legumes, (6) red and processed meat, (7) fish, (8) sodium, (9) alcohol, (10) trans-fat, and (11) long-chain fats. Each of the 11 components is scored from 1 (poor adherence) to 10 (perfect adherence), with the total AHEI-2010 diet quality score therefore ranging from 0 (non-adherence) to 110 (perfect adherence), the higher AHEI score. Further information about the AHEI-2010 and how it was collected and applied in this study can be found elsewhere (8). We based the diet quality rating categories on previous studies showing approximations (<38 points indicating a “low quality,” 3–67 points as “intermediate,” and >68 points as a “high” diet quality) (21). Diet consumption data for the AHEI-2010 components 1–9 were collected on the Australian Eating Survey and calculated using the Australian food composition databases, AusNut 2011-2013 and AusFoods Revision 5 (Australian Government Publishing Service, Canberra) over a 3-month period. Data on the AHEI-2010 compoents 10 and 11 were calculated using NUTTAB 2010 (database developed by the Food Standards Australia and New Zealand) and FoodWorks (version 18, The Nutrition Company, Long Valley, NJ, USA). Secondary outcomes, which related to participants' set goals, included fruit and vegetable intake, fiber intake, sodium intake, blood pressure, and body weight. Fruit and vegetable intake, fiber intake, and sodium intake were measured using the Australian Eating Survey. Body weight and blood pressure were collected as part of usual care where possible or by a trained site investigator who was blind to the treatment allocation, following a standardized protocol (8).

Total goal attainment was determined based on the number of “yes” responses to the weekly goal check text messages. The number of goal achievements was measured between 0 (i.e., no goal achieved) to 12 (i.e., all goals achieved) in each 12-week phase. If participants did not respond to the goal check message, these data were recorded as goal not achieved.

For statistical analysis, participant goals were categorized into either a “no goal” (received all other text messages but elected not to set a goal outside the dietary intervention targets), a “fruit and/or vegetable” goal (as the majority of participants set both fruit and vegetable intake goals, rather than one of these food groups separately), or a “healthy eating” goal. These three dietary goals were the most frequently set goals by participants with enough data available to conduct an exploratory analysis. The baseline-observation-carried-forward (BOCF) approach was applied to impute missing data in the current study. Data were checked for normality. One-way analyses of covariance (ANCOVA; adjusted for baseline) were conducted to determine potential differences in the primary and secondary outcomes differed across the two groups defined as whether participants had set one of the three listed goals or not, in each phase. Results from the analyses are presented as mean and 95% confidence interval (CI). A *p* < 0.05 (two sided) was considered statistically significant. However, given the exploratory nature of this pilot study, all results were also considered against minimal clinically important difference (MCID), which was informed by a thorough review of the literature and expert clinical guidance. For the purpose of this study, we defined MCID as 20% change in diet quality (AHEI score) (22), 5 grams (g) change in fiber intake (23), 0.5 serves in fruit intake (24), one serve in vegetable intake (24), 780 milligrams (mg) in sodium intake (25), 5 mmHg systolic blood pressure (SBP) (26), and 5% change in body weight (27). All statistical tests were performed using SPSS (version 26. *Chicago: SPSS Inc*.).

## Results

All 41 intervention participants from the previous ENTICE-CKD study were included in the analysis (63% male; mean age 63 ± 12 years; Table 2). Comorbidities were common across the population, 83% of participants having hypertension, 39% having diabetes, and 34% having cardiovascular disease.

**Table 2:** Baseline characteristics.

**Characteristic**	**Participant (*n =* 41)**
Age, years	63 ± 12
Gender, % of male	63
**Ethnicity, *n* (%)**	
Caucasian	35 (85)
Asian	2 (5)
European	2 (5)
Indigenous	1 (2.5
Other	1 (2.5)
Hypertension, *n* (%)	34 (83)
Diabetes, *n* (%)	16 (39)
Cardiovascular disease, *n* (%)	14 (34)
eGFR, ml/min/1.73 m^2^	36 ± 12
Systolic blood pressure, mmHg	136 ± 18
Diastolic blood pressure, mmHg	80 ± 12
Weight, kg	96 ± 22
Body mass index, kg/m^2^	33 ± 7
Alternate Healthy Eating Index	71.4 ± 11.8
Fruit, serves/day	1.5 ± 0.9
Vegetables, serves/day	4.2 ± 2.0
Sodium, mg	2,379 ± 1,392
Fiber, g	24.1 ± 9.1

*Data were reported as mean ± SD*.

*eGFR, estimated glomerular filtration rate; g, grams; mg, milligrams; kg, kilograms*.

At baseline (Table 2), the mean AHEI of participants was 71.4 points (rating in the “high” diet quality category). The average fruit and vegetable intake was 1.5 ± 0.9 serves and 4.2 ± 2.0 serves, respectively. The average fiber and sodium consumptions were 24.1 ± 9.1 g and 2,379 ± 1,392 mg, respectively. Participants had a mean body mass index (BMI) and SBP of 33 ± 7 kg/m^2^ and 136 ± 18 mmHg, respectively.

The most common goals set by participants included “general healthy eating” goal (*n* = 15 in phase 1; *n* = 21 in phase 2), “fruit and/or vegetable” goal (*n* = 16 in phase 1; *n* = 13 in phase 2), and “no goal” (*n* = 5 in phase 1; *n* = 7 in phase 2). A total of 944 goal check messages were sent to participants throughout the trial, 365 messages in phase 1 and 579 messages in phase 2. Participants' response rates to goal check messages were 46.3% in phase 1 and 38.7% in phase 2, respectively, of which, 41.9% of participants stated they met their set goal in phase 1 and 34% in phase 2.

Table 3 shows the effect of goal setting on diet intake. Compared with participants who did not set a “fruit and/or vegetable” goal in phase 1 (*n* = 25), those that did (*n* = 16) had a statistically significant improvement in AHEI (+6.9 points, 95% CI 1.2–12.7; 10% increase), vegetable intake (1.1 serves per day, 95% CI 0.0–2.3), and fiber intake (+4.2 g, 95% CI 0.2–8.2). Fruit intake was the only variable shown to clinically meaningfully improve (+0.5 serves, 95% CI −0.1–1.1); however, it was not statistically significant. At 6 months, these associations were all attenuated; however, a significant (but unlikely clinically meaningful) increase in sodium intake (+413.7 mg, 95% CI 28–799.4) was observed in participants setting a “fruit and/or vegetable” goal (*n* = 13) compared with those who did not.

**Table 3:** Between group changes in AHEI and dietary intake across the two groups of participants who either set a specific goal compared with participants who did not, in each phase of the study.

**Outcomes^#^Goal**	**Phase**	**AHEI (points)**	**Fruit (serves/day)**	**Vegetables (serves/day)**	**Fiber (g/day)**	**Sodium (mg/day)**
Healthy eating goal^a^	Phase 1	−0.2 (−6.3, 5.9)	−0.3 (−1.0, 0.4)	0.6 (−0.6, 1.8)	0.6 (−3.7, 4.9)	109.8 (−407.9, 627.5)
	Phase 2	−1.0 (−7.3, 5.3)	−0.2 (−0.9, 0.4)	0.3 (−0.6, 1.3)	−0.3 (−3.6, 3.0)	−210.0 (−590.1, 170.2)
Fruit and/or vegetable goal^b^	Phase 1	6.9 (1.2, 12.7)^*^	0.5 (−0.1, 1.1)	1.1 (0.0, 2.3)^*^	4.2 (0.2, 8.2)^*^	−155.6 (−659.9, 348.6)
	Phase 2	−2.0 (−9.0, 5.0)	0.0 (−0.6, 0.7)	−0.5 (−1.5, 0.6)	0.4 (−3.1, 4.0)	413.7 (28.0, 799.4)^*^
No goal^c^	Phase 1	−4.5 (−13.4, 4.3)	−0.5 (−1.4, 0.5)	−0.7 (−2.4, 1.1)	−4.0 (−10.2, 2.1)	115.2 (−643.6, 874.0)
	Phase 2	1.1 (−7.4, 9.6)	0.0 (−0.8, 0.8)	0.2 (−1.1, 1.5)	−0.0 (−4.4, 4.3)	309.1 (−191.7, 810.0)

*Data are reported as mean (95% CI)*.

a *Phase 1: “yes” n = 15; “no” n = 26. Phase 2: “yes” n = 21; “no” n = 20*.

b *Phase 1: “yes” n = 16; “no” n = 25. Phase 2: “yes” n = 13; “no” n = 28*.

c *Phase 1: “yes” n = 5; “no” n = 36. Phase 2: “yes” n = 7; “no” n = 34*.

# *Standard ANCOVA analysis reporting the mean (95% CI) change in the group of participants who set each specific goal, minus the change in participants who did not set each specific goal*.

* *p < 0.05*.

In those who set a “general healthy eating” goal (vs. those who did not), no statistically or clinically meaningful differences in any of the outcomes were observed at either 3 or 6 months. No associations were observed at 3 or 6 months in those who set “no goal” vs. those who set a goal.

There was no clinically or statistically significant effects observed for body weight or SBP (Supplementary Table 1).

## Discussion

This secondary analysis from the previous ENTICE-CKD study aimed to investigate the relationship between patient-centered goal setting and diet quality improvement and determine the specific dietary goals that might help people with CKD to improve their diet quality and clinical outcomes. Significant and potentially meaningful associations were primarily observed at 3 months. These results are exploratory and hypothesis generating and suggest that focusing on fruit and vegetable intake goals can help to promote a clinically significant short-term improvement in diet quality, vegetable and fruit intake, and dietary fiber intake with dietitian-led telehealth coaching.

Both diet quality and fiber intake are important in CKD self-management. Compared with other goal categories set by participants, “fruit and/or vegetable” goals appeared to be the strongest enabler of achieving an improved diet quality, vegetables and fiber intake in CKD patients, with the support of intensive telehealth coaching from a dietitian over 3 months. In one of the largest longitudinal analyses of continuous changes in the diet quality, a 20% improvement in AHEI was associated with an 8–17% reduced risk of all-cause mortality in people who ranked in the intermediate diet quality category (22). While the overall results of this exploratory study are far from conclusive, the AHEI improvement observed in phase 1 (+7 points; 10% improvement) is promising, particularly given the fact that our participants' baseline diet already ranked in the “high” diet quality category (22, 23). Similarly, with the noted clinical meaningful changes in fruit (0.5 serves) and vegetable intake (one serve), this placed participants mean intakes at two and five serves, respectively, in line with the Australian Dietary Guidelines (19). The fact that sodium intake increased by ~400 mg at 6 months is interesting and unexpected. It is important to note that at 3 months, sodium intake decreased by a non-significant 150 mg, in line with the significant improvement in diet quality. While we are not able to determine the exact reason why this occurred, the 6-month period of the study was typically (for the vast majority of participants) occurring over the winter and spring seasons in Australia. These seasons present well-known challenges for people with CKD in controlling their sodium intake, typically due to choosing more tinned vegetables and prepared soups (28). This also highlights the challenge that some participants had in maintaining their dietary changes after the initial 3-month coaching period, where they could not speak to a dietitian to discuss these potential issues. Future telehealth coaching studies should ensure specific education content (both telephone coaching, but likely more importantly, extended text message support), which considers assisting patients choose low-sodium pre-prepared foods and provide seasonal recipes.

Fruits and vegetables have many properties, which might make them an appealing intervention strategy for people with CKD trying to improve their diet quality. Fruits and vegetables release potassium salts, which generate bicarbonate naturally, and can decrease the kidney acid load. Current best-practice guidelines recommend that people with stages 1–4 CKD consume a diet higher in fruits and vegetables to effectively manage metabolic acidosis (4). Fruits and vegetables also have a low bioavailability of dietary phosphorus and calcium, which limits its bioavailability due to the presence of phytate in vegetable forms of phosphorous, which can also promote better adherence to nutrient-restricted interventions (29).

Adequate daily intakes of fruits, vegetables, and fiber reflect a higher overall diet quality (30). Evidence shows that sufficient fiber intake reduces the risk of inflammatory reactions, which are directly linked with the progression of kidney diseases (31). Moreover, fiber is found to be related to inflammation metabolic acidosis and gut microbiota culture, which are also associated with CKD (32). Increasing dietary fiber intake may also attenuate imbalance in the gut microbiome, which is known to have a role in modification of protein-bound uremic toxins (33). A meta-analysis of 14 CKD controlled-feeding trials showed dietary fiber to significantly reduce serum urea and creatinine levels (34). As the most common dietary sources of fiber, increasing fruit and vegetable consumptions are likely to be the most practical way in which CKD patients can improve their fiber intake and overall diet quality.

No significant AHEI improvement was found at 6 months compared with the first 3 months of the study. However, this result is not conclusive due to the low participants' response rate to goal check text messages (<50% in phase 1 and approximately one third in phase 2). While it is critical to acknowledge the message response rate, it may also be important to consider the results in the context of the differences in intervention approaches used in each phase. Specifically, in phase 1, participants received coaching contact from the dietitian with six fortnightly telephone coaching and weekly tailored text messages for the first 3 months of the program, while in the second phase of the study, participants continued receiving the same tailored text messages but received no further telephone coaching with their dietitian. It was previously reported that previous ENTICE-CKD participants needed at least two coaching calls to start putting their set action plans in place (18). According to the achievement motivation theory, people tend to continue making positive changes once they have attained their set goals (35, 36). It remains unclear whether intervention decay observed in our study is commensurate with a reduction in coaching intensity. It is likely that more intensive support over a longer duration from a dietitian is needed to promote greater change (37). An overview of the systematic reviews evaluating intervention components associated with increased effectiveness in dietary generally supports this theory, of a positive relationship between the number of intervention contacts over longer durations and self-reported dietary change (38). Whether or not this is the case specifically in CKD populations requires further investigation.

Furthermore, there were differences in the number of participants who set fruit and vegetable intake goals in each phase of the study. Specifically, in phase 2, less participants chose to continue their goal to improve their fruit and vegetable intake (*n* = 16 in phase 1 compared with *n* = 13 in phase 2). Instead, there appeared to be an increase in the number of participants who set a healthy eating goal in phase 2 compared with that in phase 1 (*n* = 15 and *n* = 21, respectively). This might indicate that participants felt that they had successfully achieved their goal to improve fruit and vegetable intake at 3 months and decided to set a more general healthy eating goal in phase 2. As mentioned above, it is likely that people with CKD still need more coaching over a longer period as mentioned. However, it could also be hypothesized that people with CKD may need more specific goals, such as a fruit or vegetable intake goals, compared with a general healthy eating goal, in order to achieve true change in diet quality. This hypothesis needs testing in future adequately powered randomized controlled trials.

This study has important limitations to consider. This is a *post-hoc* secondary analysis, which only evaluated a single arm of the previous ENTICE-CKD trial with no control group comparison. The findings in this study are therefore exploratory only, hypothesis-generating, and not conclusive. The small sample size is a factor influencing the accuracy of results. Participants showed diverse preferences for dietary goals throughout the previous ENTICE-CKD program, in line with the practical and patient-centered approach the intervention was underpinned by. While most participants determined that “healthy eating” and “fruit and/or vegetable” goals were important to them (*n* = 13 and *n* = 21, respectively), very few participants set goals such as a “weight reduction” goal (*n* = 2), and these goals could not be analyzed in a reliable or meaningful way. This limited our ability to explore the effect of specific goal setting on improving clinical outcomes. Participants showed a “high” diet quality score at baseline indicating healthy volunteer bias; this limitation may underestimate the potential benefits of dietary intervention evaluated in this study. To manage the issue of missing data, the principle of BOCF was applied to impute missing data, which may underestimate the treatment effect, or may ignore the possibility that people could regress or even worsen from baseline. Finally, while this is a patient-centered approach, our study relies almost entirely on self-reported data, which may reduce the confidence in the conclusions inferred.

In conclusion, patient-centered goal setting, which focuses on fruit and vegetable intake appeared to significantly improve diet quality, fruit and vegetable intake, and fiber intake in people with stages 3–4 CKD. No significant results were found at 6 months, which may be due to the reduction of coaching intensity and suggests that a longer-term telehealth coaching may be required in helping CKD patients to achieve long-term self-management. Further research evaluating longer-term interventions, with an emphasis on goal setting particularly around fruit and vegetable intake, will determine what effect on clinical outcomes are possible for people with CKD.

## Data Availability Statement

The raw data supporting the conclusions of this article will be made available by the authors, upon reasonable request and clear and robust scientific protocol provided, without undue reservation.

## Ethics Statement

The research protocol was approved by Metro South Hospital and Health Service and Bond University Human Research Ethics Committees. The patients/participants provided their written informed consent to participate in this study.

## Author Contributions

CC completed the data analysis and wrote the first draft of the manuscript. JK is the supervisor of this study who contributed to the study conception, original research data collection, reviewed the analysis results, and critically reviewed versions of the manuscript. MC participated in the original data collection and intervention design. MR and KC contributed to the study conception and critically reviewed the manuscript. All authors critically reviewed the manuscript and approved the final version of the manuscript.

## Conflict of Interest

The authors declare that the research was conducted in the absence of any commercial or financial relationships that could be construed as a potential conflict of interest.
