# Supplementary material for: Evaluating the Impact of Goal Setting on Improving Diet Quality in Chronic Kidney Disease
Source: Front Nutr. 2021 Mar 12;8:627753. doi: 10.3389/fnut.2021.627753 (PMC7994896; doi:10.3389/fnut.2021.627753)
Supplement: Supplementary file 1 [file Table_1.DOCX]

**Supplemental Table 1:** Between group changes in weight and systolic blood pressure across the two groups of participants who either set a specific goal compared to participants who did not, in each phase of the study. Data are reported as mean (95% CI).

| **Outcomes^#^**  **Goal** | **Phase** | **Weight (kg)** | **Systolic blood pressure (mmHg)** |
| --- | --- | --- | --- |
| **Healthy Goal^a^** | Phase 1 | -0.1 (-1.9, 1.8) | 1.7 (-7.0, 10.3) |
|  | Phase 2 | -0.9 (-3.5, 1.8) | -1.6 (-12.9, 9.7) |
| **Fruit and/or Vegetable Goal**^b^ | Phase 1 | -1.3 (-3.0, 0.5) | -2.9 (-11.3, 5.5) |
|  | Phase 2 | 0.0 (-2.9, 2.9) | -3.8 (-15.9, 8.4) |
| **No Goal^c^** | Phase 1 | 1.7 (-1.0, 4.4) | 0.9 (-11.7, 13.6) |
|  | Phase 2 | 1.0 (-2.5, 4.5) | 6.5 (-8.4, 21.3) |

^a^ Phase 1: ‘yes’ n=15; ‘no’ n=26. Phase 2: ‘yes’ n=21; ‘no’ n=20.

^b^ Phase 1: ‘yes’ n=16; ‘no’ n=25. Phase 2: ‘yes’ n=13; ‘no’ n=28.

^c^ Phase 1: ‘yes’ n=5; ‘no’ n=36. Phase 2: ‘yes’ n=7; ‘no’ n=34.

**^#^** Standard ANCOVA analysis reporting the mean (95% CI) change in the group of participants who set each specific goal, minus the change in participants who did not set each specific goal.
